# The systematic position of Dryopteris blanfordii subsp. nigrosquamosa (Ching) Fraser-Jenkins within the genus *Dryopteris* Adans.

**DOI:** 10.3897/phytokeys.90.14745

**Published:** 2017-11-15

**Authors:** Anastasiya A. Krinitsina, Maxim S. Belenikin, Olga A. Churikova, Sergey V. Kuptsov3, Maxim I. Antipin, Maria D. Logacheva, Anna S. Speranskaya

**Affiliations:** 1 Department of High Plants, Biological Faculty, Lomonosov Moscow State University, Leninskie gory, 1, 12, Moscow, Russia, 119234; 2 Department of Molecular and Biological Physics, Moscow Institute of Physics and Technology, Dolgoprudny, Moscow Region, Russia, 141700; 3 Botanical Garden, Lomonosov Moscow State University, Leninskie gory, Moscow, Russia, 119899; 4 Department of Evolutional Biochemistry, A.N. Belozersky Institute of Physico-Chemical Biology, Lomonosov Moscow State University, Leninskie gory, 1, 40, Moscow, Russia, 119992

**Keywords:** chloroplast genome markers, Dryopteridaceae, *Dryopteris blanfordii subsp. nigrosquamosa*, phylogeny

## Abstract

*Dryopteris blanfordii* (C.Hope) C.Chr. is a member of the Dryopteridaceae, growing in high altitude *Picea* or *Abies* forests (2900–3500 m) in China and India. Phylogenetic relationships between D. blanfordii subsp. nigrosquamosa and closely related species of *Dryopteris* were investigated using a combined analysis of multiple molecular data sets (the protein-coding region of *rbcL* and *matK* genes and intergenic spacers *psbA-trnH*, *trnP-petG*, *rps4-trnS*, *trnL-trnF* and *rbcL-accD*). An assumption about the position of D. blanfordii subsp. nigrosquamosa within *Dryopteris* was made by using the Maximum Likelihood and Bayesian Inference approach and chloroplast marker sequences of *Dryopteris* species from GenBank. The results demonstrated that Asian taxa D. blanfordii subsp. nigrosquamosa and *D. laeta* as well as two American species *D. arguta* and *D. marginalis* belong to the same clade, all four of them being part of Dryopteris section Dryopteris.

## Introduction

*Dryopteris* is a large fern genus of some 225–230 species belonging to the Dryopteridaceae (Fraser-Jenkins 1986, Kramer 1990, Zhang et al. 2012). *Dryopteris blanfordii* (C.Hope) C.Chr. grows in *Picea* or *Abies* forests at 2900–3500 m in China (Gansu, Sichuan, Xizang, Yunnan), Afghanistan, India, Kashmir, Nepal and Pakistan (Zhang et al. 2013). It is divided into two subspecies, *nigrosquamosa* (Ching) Fraser-Jenkins and *blanfordii*. Dryopteris blanfordii subsp. nigrosquamosa is abundant in India (the Kashmir valley) (Mir et al. 2014, Mir et al. 2015), China (Gansu, Sichuan, Xizang, Yunnan) and Nepal (Zhang et al. 2013).

The current taxonomy and infrageneric position of *D. blanfordii* are still unresolved. More than a quarter of a century ago, Fraser-Jenkins (1986) divided *Dryopteri*s into several sections based on the comparison of macro- and micro-morphological traits. He established Sect. Remotae with a single species (*D. blanfordii*) in the Indian subcontinent and with two European species (*Dryopteris remota* Hayek. and *D. corleyi* Fraser-Jenk.). Fraser-Jenkins suggested that all species from Sect. Remotae are allopolyploids resulting from hybridization between species from different sections (such as sect. Fibrillosae, sect. Lophodium or sect. Marginatae) (Fraser-Jenkins 1986).

In a recent study, a phylogeny including 100 species of *Dryopteris* was reconstructed and 13 phylogenetic clades (or major evolutionary lineages) were identified using DNA sequences of four plastid loci (*rbcL* gene, *rps4-trnS* spacer, *trnL* intron and *trnL-F* spacer) (Zhang et al. 2012). Two of the three species, previously placed in Sect. Remotae, were identified in this study as members of other clades, namely clade Aemulae (*D. corleyi*) and clade Lophodium (*D. remota*) (Zhang et al. 2012). Similar results were demonstrated by Sessa et al. (2012a, 2012b), where seven plastid loci (*rbcL*, *psbA-trnH*, *trnP-petG*, *rps4-trnS*, *trnL-F*, *trnG-trnR* and *rbcL-accD*) and a single nuclear marker *pgiC* were used to analyse 97 *Dryopteris* species. The data of these phylogenies, although valuable, were far from complete and the taxonomic position of D. blanfordii subsp. nigrosquamosa remained unclear.

## Material and methods

### Plant material

To examine the morphology of D. blanfordii subsp. nigrosquamosa, adult plants from the Botanical Garden of Moscow State University were used. The parent plant of Dryopteris blanfordii (C.Hope) C.Chr. subsp. nigrosquamosa (Ching) Fraser-Jenk was collected in 2003 in Uttar Pradesh State, India, at 3000 m. Spores of the specimen were germinated under artificial conditions in the greenhouse complex of MSU Botanical Garden. Subsequently, developed sporophytes were transplanted to the outdoor section of the Botanical Garden. The adult specimens were used for DNA sampling. The voucher specimen was deposited at Herbarium MW. Reference morphological characters for D. blanfordii subsp. nigrosquamosa were scored from a type specimen (PE 00133945, locality: Tibet) and from descriptions of Dryopteris blanfordii (C.Hope) C.Chr. subsp. nigrosquamosa (Zhang et al. 2013, Mir et al. 2014, Mir et al. 2015).

### Chloroplast markers sequencing and assembling

The chloroplast marker sequences of D. blanfordii subsp. nigrosquamosa were obtained during a large project on Polypodiales chloroplast genome sequencing. Sequencing data were generated using Illumina MiSeq high-throughput sequencing platform. For sample preparation, adult living plants were taken from the collection of the MSU Botanical Garden. The cpDNA fraction was extracted from 2.6 g (fresh weight) of fronds using a slightly modified cpDNA extraction protocol (Shi et al. 2012, Vieira et al. 2014). The purification of DNA was carried out using a protocol designed by the authors (Krinitsina et al. 2015). TruSeq protocol (NEBNext® DNA Library Prep Master Mix Set for Illumina, E6040, NEB reagents) was used for preparing the libraries. Pare end (PE) sequences (2×300bp) with a double number of each library reads about 1.2–1.97M were made. After quality trimming by Trimmomatic (Bolger et al. 2014), reads were filtered using 13 complete and five partial fern chloroplast genome sequences from RefSeq database and Bowtie2 (Langmead and Salzberg 2012). Then two sets of contigs were produced for both filtered and unfiltered sets of reads using Velvet Assembler (Zerbino and Birney 2008) and MIRA4 (Chevreux et al. 2004). Assembled contigs and scaffolds were used for assembling the complete chloroplast genome (the data are not presented in this paper) and for extracting target chloroplast markers, namely *rbcL, matK* genes and intergenic spacers *psbA-trnH*, *trnP-petG*, *rps4-trnS*, *trnL-trnF* and *rbcL-accD*.

### Dryopteris blanfordii subsp. nigrosquamosa phylogenetic analysis

To determine the phylogenetic position of D. blanfordii subsp. nigrosquamosa, a phylogenetic analysis using sequences published in GenBank was performed. The GenBank accession numbers of sequences of *Dryopteris* species included in this study are listed in Appendix 1. Sequence alignment was conducted using Muscle algorithm and MEGA6.0 software package (www.megasoftware.net, Tamura et al. 2013). Phylogenetic analyses were performed using the Maximum Likelihood (ML) method at MEGA 6.0 (Tamura et al. 2013) and Bayesian Inference (BI) in BEAST (Bouckaert et al. 2014). A combined matrix including *matK* and *rbcL* gene and five intergenic spacers (*psbA-trnH*, *rps4-trnS*, *trnL-trnF trnP-petG* and *rbcL-accD*) of 84 *Dryopteris* species (including D. blanfordii subsp. nigrosquamosa) was analysed.

A bootstrapping of 1000 replicates for ML analysis was processed to estimate the confidence probabilities on each branch of the phylogenetic trees constructed. The initial tree (ML) for heuristic search was obtained by applying the Neighbour-Joining method to a matrix of pairwise distances estimated using the Maximum Composite Likelihood approach (Lindsay 1988, Tamura et al. 2013). All positions containing gaps and missing data were eliminated.

Bayesian analyses were run for 20,000,000 generations with four MCMC chains in two independent runs. The first 2,000,000 samples from each run were discarded as burn-in. Convergence was assessed by comparing the standard deviation of split frequencies between different runs (MCMC Trace Analysis Tool (Tracer) version v1.6.0 (Rambaut et al. 2014). For ML and BI analyses, optimal models of molecular evolution for combined matrices were identified using jModelTest2 (Darriba et al. 2012) through Bayesian Information Criterion (BIC).

## Results

### Dryopteris blanfordii subsp. nigrosquamosa phylogenetic position

Seven marker regions of the assembled cp genome were used for determining phylogenetic relationships between D. blanfordii subsp. nigrosquamosa and other *Dryopteris* species, i.e. protein-coding regions of *rbcL* and *matK* genes and intergenic spacers *psbA-trnH*, *trnP-petG*, *rps4-trnS*, *trnL-trnF* and *rbcL-accD*. These markers were assembled into a single data matrix consisting of 3734 total bases. The optimal model of molecular evolution for combined matrices was TPM1uf+G+I with BIC = 36592.7258. The phylogenetic tree is shown in Fig. 2. The analysis demonstrated close relationships between D. blanfordii subsp. nigrosquamosa, *D. laeta, D. marginalis* and *D. arguta*. The clades containing D. blanfordii subsp. nigrosquamosa were well-supported (≥80% bootstrap support). Dryopteris blanfordii subsp. nigrosquamosa is close to *D. laeta* (bootstrap=100/PP=100%), *D. arguta* (bootstrap=87/PP=99.6%) and *D. marginalis* (bootstrap=96/PP=100%). The results of Bayesian Inference analysis based on the combined matrix were highly congruent with the strict consensus tree from ML analysis. The clade that included *D. arguta, D. marginalis*, *D. laeta* and D. blanfordii subsp. nigrosquamosa in the combined matrix of seven markers had the posterior probability (PP) value of 100%.

### Morphological characters of D. blanfordii subsp. nigrosquamosa and closely related species.

**Figure 1.**
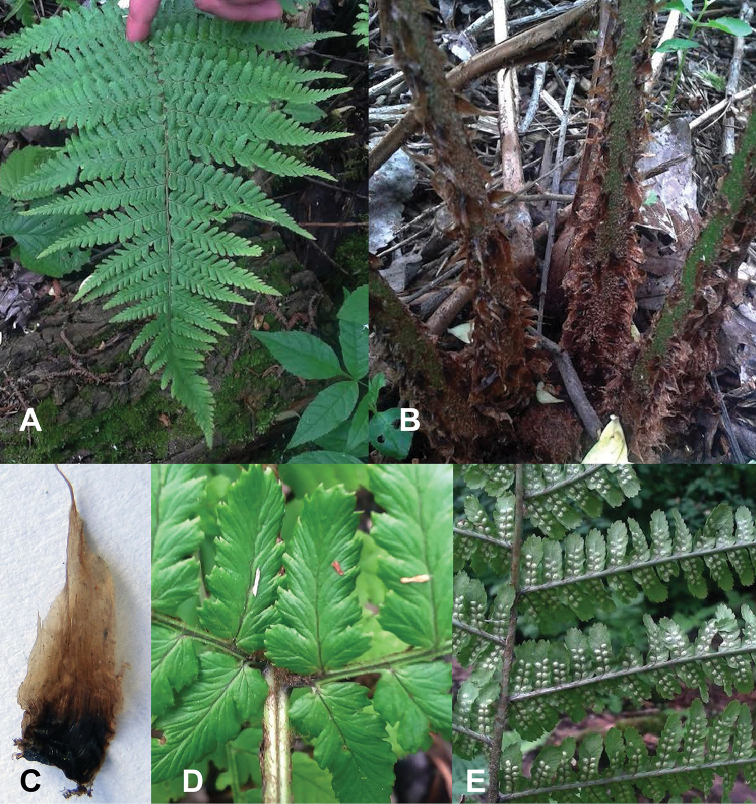
Dryopteris blanfordii subsp. nigrosquamosa morphology. **A** Frond of mature plants **B** Petiole covered with scales **C** Petiole scale **D** Adaxial surface of rachis and costa **E** Abaxial surface of rachis and costa.

The adult specimens of *D. blanfordii* analysed in the present work have 55–57×30–35cm fronds. The frond dissection is 2-pinnate with symmetrical pinnae and pinnules (Fig. 1A). The rachises and petioles are fibrillose and have dense basal scales. The scales on the petioles are dark-brown basally and light-brown at the apex (Fig. 1 B and C). The costa and rachises are slightly grooved adaxially (Fig. 1D) and rounded abaxially (Fig. 1E).

Two closely related species, namely *D. arguta* and *D. marginalis*, are native to North America. *Dryopteris marginalis* is evergreen and has tawny or cinnamon-coloured scales, lanceolate and coriaceous laminae, with sori mostly at margins of ultimate pinnules’ segments (Table 1). *Dryopteris arguta* is winter green, having grassy-green to yellow-green, ovate-lanceolate, herbaceous, glandular laminae; the basal basiscopic pinnule and basal acroscopic pinnule are ± equal; its pinnule margins are serrate with spreading, spinelike teeth; the sori are medial. Both North American species have longer stipes (1/4–1/3 length of leaf), 1-pinnate-pinnatifid to 2-pinnate-pinnatifid fronds (Montgomery and Wagner 1993).

**Table 1.** Morphological characters of four species of *Dryopteris*: D. blanfordii subsp. nigrosquamosa, *D. laeta*, *D. marginalis* and *D. arguta* according to Montgomery and Wagner (1993), Mir et al. (2014), Mir et al. (2015).

Species	D. blanfordii subsp. nigrosquamosa	*D. laeta*	*D. marginalis*	*D. arguta*
Natural range	Southeast Tibet, Western China, Nepal, India (Kashmir)	North China, Eastern Siberia, North Korea and North Japan	Eastern North America	From British Columbia to Baja California
Seasonality	semi-evergreen	deciduous	evergreen	winter green
Rhizomes	erect	creeping	ascending to erect	short-creeping
Scale colour	light brown with black veins	pale brown	tawny to cinnamon	light brown
Lamina length (cm)	40–75	25–50	25–50 (75)	25–90
Lamina division	2-pinnate to 3-pinnate-pinnatifid	3-pinnate-pinnatifid	1-pinnate-pinnatifid to 2-pinnate-pinnatifid	2-pinnate-pinnatifid
Lamina colour and texture	glaucous green, coriaceous	green, herbaceous to thinly papyraceous	green, coriaceous	green to yellow-green, herbaceous, glandular
Lamina shape	lanceolate to oblong-lanceolate	ovate-oblong or deltoid-ovate	ovate-lanceolate	ovate-lanceolate
Stipe length	1/5-1/4 of rachis length	1/3 to 1/2 of rachis length	1/4 to 1/3 of rachis length	1/4 to 1/3 of rachis length
Sori arrangement	in 1 row at each side of midvein, inframedial	in 2 rows at each side of midvein	in 1 row at each side of midvein, intramarginal at margins of segments	in 1 row at each side of midvein, medial

*Dryopteris laeta* is characterised by a long stipe (length roughly equal to blade length) with very few lanceolate scales; deciduous, ovate-oblong or deltoid-ovate, 3-pinnate-pinnatifid, 25–50×15–40cm, herbaceous to thinly papyraceous laminae; pinnules with toothed margins ending in an acute apex; sori in 1 or 2 rows on each side of pinnule costa; indusia orbicular-reniform, membranaceous, margin eroded (Zhang et al. 2013). The main morphological characters of these four species of *Dryopteris* are presented in the table below.

## Discussion

**Figure 2.**
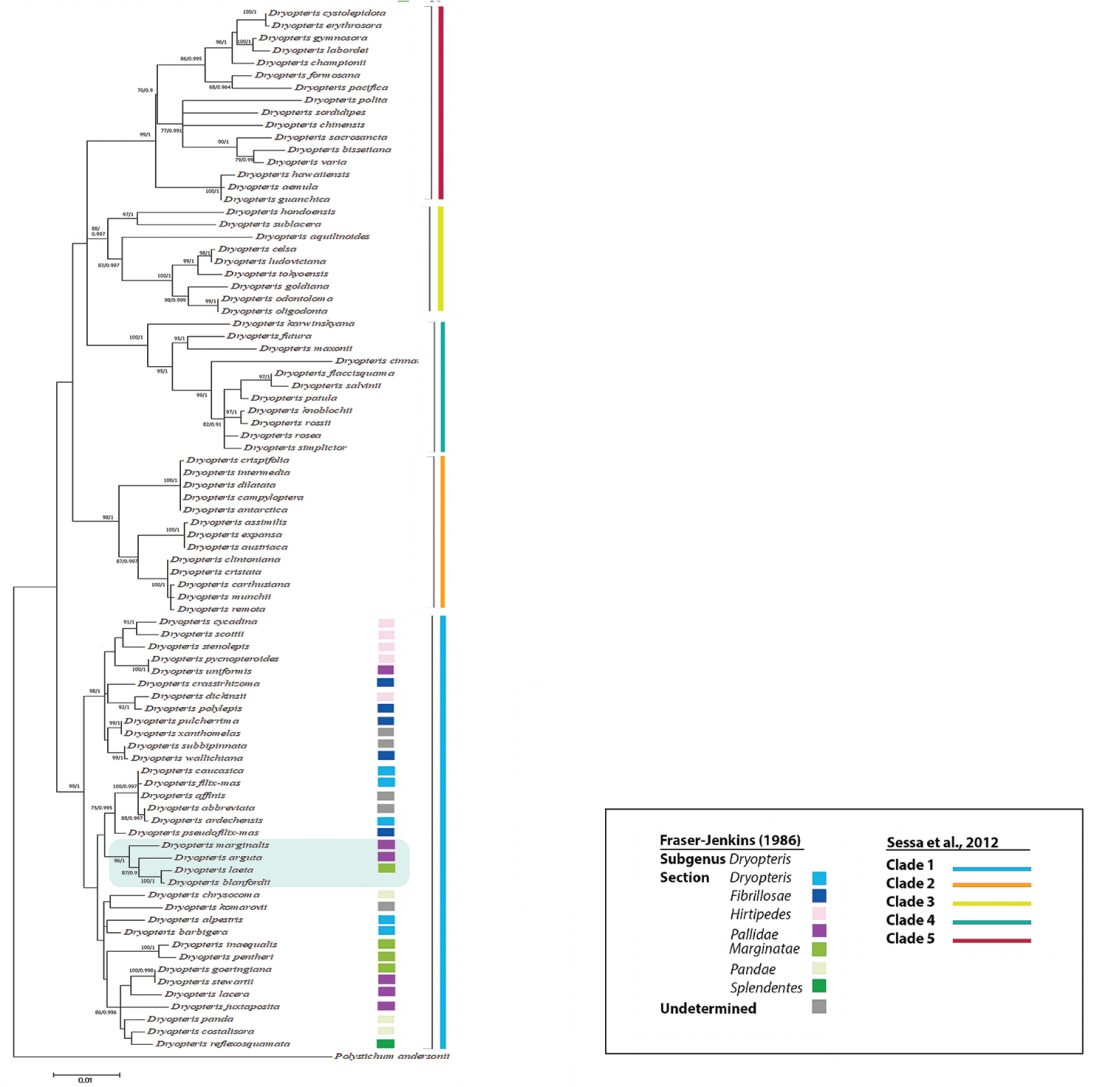
Topology from ML and BI analyses of *Dryopteris* species using six marker regions consisting of 3734 total bases (*psbA-trnH*, *rbcL-accD, rbcL*, *trnL-trnF, trnP-petG*, *rps4-trnS and matK*). The tree is drawn to scale, with branch lengths measured in the number of substitutions per site. Bootstrap values are specified at the branch nodes (cut off >50%) / Bayesian РР. Dryopteris blanfordii subsp. nigrosquamosa and closely related species are marked with a blue rectangle.

The data obtained in this study allowed us to suggest a more accurate view of the taxonomic position of D. blanfordii subsp. nigrosquamosa. Our results demonstrated that *D. laeta* and D. blanfordii subsp. nigrosquamosa belong to the same clade as *D. arguta* and *D. marginalis*. According to the classification system of the genus *Dryopteris* by Fraser-Jenkins (1986), *D. arguta* and *D. marginalis* belong to sect. Pallidae, while *D. blanfordii* belongs to sect. Remotae. More recent classifications divide the genus *Dryopteris* into either five (Sessa et al. 2012b) or 13 different clades (Zhang et al. 2012). We have concluded that D. blanfordii subsp. nigrosquamosa together with *D. laeta*, *D. arguta* and *D. marginalis* belong to the *Dryopteris* clade following Zhang et al. (2012) or clade I according to Sessa et al. (2012b). *Dryopteris arguta* and *D. marginalis* are closely related American species (from western and eastern parts of North America respectively) and the *D. laeta* specimen is from a population located in Iwaizumi (Iwate prefecture, Japan). According to some recent studies (Widén et al. 2015), *Dryopteris goeringiana* (Kunze) Koidz. is a synonym of *D. laeta*, growing in Japan. Our analyses rather showed that *D. goeringiana* proves to be related to *Dryopteris stewartii* Fraser-Jenk., *Dryopteris lacera* (Thunb.) Kunze and *Dryopteris sieboldii* (Van Houtte ex Mett.) Kuntze (Fig. 2), which agrees with the data of other authors (Sessa et al. 2012b). At the same time, *D. laeta* from Japan does not belong to this group of *Dryopteris* species (Ebihara 2011, Zhang et al. 2012) (Fig. 2). The relationship between species from Europe, Central America and Asia may indicate that a long-distance dispersal event occurred. Unfortunately, it is impossible to indicate where the ancestor of these species might have originated.

*Dryopteris arguta*, *D. marginalis*, *D. laeta* and D. blanfordii subsp. nigrosquamosa differ from each other in many morphological characters (Table 1), but share the structure of segments of second and third orders. The second order segments (pinnules) are short- or very short-stalked (1–5(10) mm). The distal part of the pinnule is attenuated and elongated, with its distal third alate and lacking sori. The basal basiscopic to acroscopic pinnulae length ratio is 1–1.5. The third order segments, when present, are isomorphic. Pinnules are serrate, ending with more or less prominent spiny teeth.

## Conclusion

The relationship of D. blanfordii subsp. nigrosquamosa within the *Dryopteris* genus was defined using phylogenetic analyses based on chloroplast markers. Our results demonstrate that Asian species D. blanfordii subsp. nigrosquamosa and *D. laeta* belong to the same clade as two North American species *D. arguta* and *D. marginalis*, with all four species being part of the Dryopteris section Dryopteris.

## Appendix 1

**Table:** GenBank accession numbers of marker sequences of *Dryopteris* species included in the study. All sequences of D. blanfordii subsp. nigrosquamosa were newly generated in this study.

Species	*matK* GenBank accession/ voucher or isolate	*psbA*-*trnH* GenBank accession / voucher or isolate	*rbcL*-*accD* GenBank accession / voucher or isolate	*rps4*-*trnS* GenBank accession / voucher or isolate	*trnL*-*trnF* GenBank accession / voucher or isolate	*trnP*-*petG* GenBank accession / voucher or isolate	*rbcL* GenBank accession / voucher or isolate
***Dryopteris abbreviata* Kuntze**		***JN189448.1*** / isolate E335	***JN189664.1*** / isolate E335	***JN189231.1*** / isolate E335	***JN189126.1*** / -	***JN189342.1*** / isolate E335	***JN189557.1*** / isolate E335
***Dryopteris aemula* (Ait.) Kuntze**		***JN189407.1*** / isolate E113	***JN189625.1*** / isolate E113	***JN189189.1*** / isolate E113	***AY268816.1*** / -	***JN189301.1*** / isolate E113	***AY268881.1*** / -
***Dryopteris affinis* (Lowe) Fraser-Jenk.**		***JN189408.1*** / isolate E114	***JN189626.1*** / isolate E114	***JN189190.1*** / isolate E114	***AY268780.1*** / -	***JN189302.1*** / isolate E114	***AY268849.1*** / -
***Dryopteris alpestris* Tagawa ex Ching & S.K.Wu**	JQ941627.1/ Heng 32147 (UC)	***JN189428.1*** / isolate E314	***JN189645.1*** / isolate E314	***JN189210.1*** / isolate E314	***JX535868.1*** / Xiaohua Jin & Liang Zhang 11103 (CDBI)	***JN189322.1*** / isolate E314	***JX535858.1*** / Xiaohua Jin & Liang Zhang 11103 (CDBI)
***Dryopteris antarctica* (Baker) C.Chr.**	JQ941648.1/ Hennequin 2009-R109 (REU)	***JN189467.1*** / isolate E378	***JN189682.1*** / isolate E378	***JN189250.1*** / isolate E378	***JN189141.1*** / isolate E378	***JN189356.1*** / isolate E378	***JN189577.1*** / isolate E378
***Dryopteris aquilinoides* (Desv.) C.Chr.**	JQ941617.1/ Kessler 13855 (UC)	***JN189429.1*** / isolate E315	***JN189646.1*** / isolate E315	***JN189211.1*** / isolate E315	***JN189106.1*** / isolate E315 AY268803.1/ Ranker 1536	***JN189323.1*** / isolate E315	***JN189537.1*** / isolate E315 AY268868.1/ -
***Dryopteris arguta* (Kaulf.) Maxon**	JQ941647.1/ EBS 35 (WIS) JQ941660.1/ EBS 36 (WIS)	***JN189400.1*** / isolate E069	***JN189619.1*** / isolate E069	***JQ936838.1*** / EBS 36 (WIS)	***AY278397.1*** / -	***JN189294.1*** / isolate E069	***JN189509.1*** / isolate E069 JQ935258.1/ EBS 36 (WIS)
***Dryopteris ardechensis* Fraser-Jenk.**		***JN189487.1*** / isolate NH56	***JN189702.1*** / isolate NH56	***JN189271.1*** / isolate NH56	***AY268817.1*** / -	***JN189377.1*** / isolate NH56	***JN189596.1*** / isolate NH56
***Dryopteris assimilis* S.Walker**	JQ941622.1/ Skvortsov 1.VIII.1982 (NY)	***JN189409.1*** / isolate E117	***JN189627.1*** / isolate E117	***JN189191.1*** / isolate E117	***JN189086.1*** / isolate E117	***JN189303.1*** / isolate E117	***JN189517.1*** / isolate E117
***Dryopteris austriaca* (Jacq.) Woyn. ex Schinz & Thell.**	JQ941637.1/ Degn 25 (NY)	***JN189410.1*** / isolate E119	***JN189628.1*** / isolate E119	***JN189192.1*** / isolate E119	***JN189087.1*** / isolate E119	***JN189304.1*** / isolate E119	***JN189518.1*** / isolate E119
***Dryopteris barbigera* (Moore) Kuntze**		***JN189431.1*** / isolate E317	***JN189647.1*** / isolate E317	***JN189213.1*** / UC<USA-CA> Miehe 94-191-14	***JN189108.1*** / isolate E317	***JN189325.1*** / isolate E317	***JN189539.1*** / UC<USA-CA> Miehe 94-191-14
***Dryopteris bissetiana* (Baker) C.Chr.**		***AB575740.1*** / TNS:763335	***JN189693.1*** / isolate NH28	***DQ191829.1*** / SG Lu/C75 JN189261.1/ isolate NH28	***AY268796.1*** / R. Moran, COLO	***JN189367.1*** / isolate NH28	***AY268862.1*** / -
***Dryopteris campyloptera* (Kunze) Clarkson**	JQ941638.1/ EBS 22 (WIS) JQ941619.1/ EBS 19 (WIS)	***JN189395.1*** / isolate E058	***JN189614.1*** / isolate E058	***JQ936819.1*** / EBS 19 (WIS)	***AY268801.1*** / WJ Cody 23484 FR731970.1/ isolate A183	***JN189289.1*** / isolate E058	***JQ935255.1*** / EBS 19 (WIS) AY268866.1/ -
***Dryopteris carthusiana* (Vill.) H.P.Fuchs**	JQ941634.1/ EBS 42 (WIS) JQ941655.1/ EBS 43 (WIS) JQ941653.1/ EBS 7 (WIS) JQ941640.1/ EBS 41 (WIS)	***JN189402.1*** / isolate E075	***JN189621.1*** / isolate E075	***JN189184.1*** / isolate E075 JQ936839.1/ EBS 42 (WIS)	***AY268777.1*** / GW Argus 9327, COLO	***JN189296.1*** / isolate E075 JQ683076.1/ EBS 43 (WIS) JQ683064.1/ EBS 7 (WIS)	***JN189511.1*** / isolate E075 JQ935266.1/ EBS 7 (WIS) JQ935272.1/ EBS 43 (WIS)
***Dryopteris caucasica* (A.Braun) Fraser-Jenk. & Corley**	JQ941604.1/ Christenhusz 4309 (UC)	***JN189432.1*** / isolate E318	***JN189648.1*** / isolate E318	***JN189214.1*** / isolate E318	***JN189109.1*** / isolate E318	***JN189326.1*** / isolate E318	***JN189540.1*** / isolate E318
***Dryopteris celsa* (W.Palmer) Small**	JQ941652.1/ EBS 27 (WIS) JQ941658.1/ Price 94-2 (NY)	***JQ936652.1*** / Price 94-2 (NY)	***JN189609.1*** / isolate E043	***JN189175.1*** / isolate E043 JQ936822.1/ EBS 49 (WIS)	***JN105314.1*** / EBS49 (WIS)	***JQ683075.1*** / Price 94-2 (NY)	***JQ935249.1*** / EBS 49 (WIS)
***Dryopteris championii* (Benth.) C.Chr.**		***JN189480.1*** / isolate NH31 AB575742.1/ TNS:764357	***JN189694.1*** / isolate NH31	***DQ151856.1*** / SG Lu/M3	***AY268797.1*** / R. Moran, COLO KC896581.1/ isolate CHA 1419	***JN189368.1*** / isolate NH31	***AY268863.1*** / - KC896547.1/ isolate CHA 1419
***Dryopteris chinensis* Koidz.**		***AB575743.1*** / TNS:763933	***JN189649.1*** / isolate E319	***JN189215.1*** / isolate E319 JX535819.1/ Liang Zhang & Zhangming Zhu 1114 (CDBI)	***JX535872.1*** / Liang Zhang & Zhangming Zhu 1114 (CDBI)	***JN189327.1*** / isolate E319	***AB575119.1*** / TNS:763933
***Dryopteris chrysocoma* (Christ) C.Chr.**		***JN189434.1*** / isolate E320	***JN189650.1*** / isolate E320	***JN189216.1*** / isolate E320 DQ191832.1/ SG Lu/C76	***DQ514495.1*** / LJM079	***JN189328.1*** / isolate E320	***DQ508773.1*** / -
***Dryopteris cinnamomea* C.Chr.**		***JN189420.1*** / isolate E279	***JN189638.1*** / isolate E279	***JN189202.1*** / isolate E279	***AY278398.1*** / - FR731991.1/ A190	***JN189314.1*** / isolate E279	***JN189528.1*** / isolate E279
***Dryopteris clintoniana* (D.C.Eaton) Dowell**	JQ941605.1/ EBS 16 (WIS) JQ941626.1/ EBS 8 (WIS)	***JQ936651.1*** / EBS 8 (WIS) JN189389.1/ isolate E020	***JN189608.1*** / isolate E020	***JN189174.1*** / isolate E020 JQ936813.1/ EBS 8 (WIS)	***JQ683004.1*** / EBS 8 (WIS)	***JN189283.1*** / isolate E020	***JQ935247.1*** / EBS 8 (WIS) KF186502.1/ OAC 96815
***Dryopteris crispifolia* Rasbach, Reichst. & G.Vida**	***JQ941650.1*** / BPSSE	***JN189488.1*** / isolate NH58	***JN189703.1*** / isolate NH58	***JN189272.1*** / isolate NH58	***AY268819.1*** / -	***JN189378.1*** / isolate NH58	***AY268884.1*** / -
***Dryopteris costalisora* Tagawa**		***JN189493.1*** / isolate NH85	***JN189710.1*** / isolate NH85	***JN189278.1*** / isolate NH85	***JN189170.1*** / isolate NH85	***JN189384.1*** / isolate NH85	***JN189603.1*** / isolate NH85
***Dryopteris crassirhizoma* Nakai**		***AB575746.1*** / TNS:764333	***JN189651.1*** / isolate E321	***JN189217.1*** / isolate E321	***AY268805.1*** / - AY278399.1/ -	***JN189329.1*** / isolate E321	***JN189543.1*** / isolate E321 AY268870.1/ - KC896537.1/ isolate CRA 176
***Dryopteris cristata* (L.) A.Gray**	JQ941613.1/ EBS 58 (WIS) JQ941608.1/ EBS 68 (WIS) JQ941614.1/ Montgomery 07-99 (NY) JQ941609.1/ Leoschke 2119 (NY) JQ941621.1/ ESB 26 (WIS)	***JQ936659.1*** / EBS 68 (WIS) JQ936654.1/ Leoschke 2119 (NY)	***JQ947928.1*** / EBS 58 (WIS)	***JQ936835.1*** / Montgomery 07-99 (NY)	***JQ682997.1*** / EBS 68 (WIS)	***JN189330.1*** / isolate E322 ***JQ683056.1***/ Montgomery 07-99 (NY) JN189299.1/ isolate E089 JQ683055.1/ ESB 26 (WIS) JQ683065.1/ EBS 68 (WIS) JQ683070.1/ EBS 58 (WIS) JQ683073.1/ EBS 52 (WIS) JQ683078.1/ Leoschke 2119 (NY)	***JQ935251.1*** / Montgomery 07-99 (NY) KF186503.1/ OAC 96818
***Dryopteris cycadina* (Franch. & Sav.) C.Chr.**		***JN189436.1*** / isolate E322	***JN189652.1*** / isolate E322	***JN189218.1*** / isolate E322 DQ191835.1/ SG Lu/C38	***JN189113.1*** / isolate E322 AY278400.1/ -	***JN189330.1*** / UC<USA-CA> RBCTW-078	***JN189544.1*** / isolate E322 EF463127.1/ - AY587115.1/ -
***Dryopteris cystolepidota* (Miq.) C.Chr.**		***JN189485.1*** / isolate NH52	***JN189699.1*** / isolate NH52	***JN189268.1*** / isolate NH52	***AY268813.1*** / -	***JN189374.1*** / isolate NH52	***JN189593.1*** / isolate NH52 AY268878.1/ -
***Dryopteris dilatata* (Hoffm.) A.Gray**	JQ941646.1/ Hennequin 2010-B1 (P) JQ941599.1/ Schuettpelz 535 (DUKE) JQ941645.1/ Camoletto 2021 (NY)	***JN189465.1*** / isolate E375 JQ936648.1/ Schuettpelz 535 (DUKE) JQ936650.1/ Camoletto 2021 (NY)	***JN189680.1*** / isolate E375	***JQ936841.1*** / Schuettpelz 535 (DUKE)	***JQ683001.1*** / Schuettpelz 535 (DUKE) AY268779.1/ Kasnoborov 679, COLO	***JN189354.1*** / isolate E375	***JN189575.1*** / - AY268848.1/ - JQ935276.1/ Schuettpelz 535 (DUKE)
***Dryopteris dickinsii* (Franch. & Sav.) C.Chr.**		***JN189489.1*** / isolate NH59	***JN189704.1*** / isolate NH59	***JN189273.1*** / isolate NH59 DQ191839.1/ SG Lu/QC3	***JX535875.1*** / K. Ohora s.n.; VS766491 (TNS) AY268820.1/ -	***JN189379.1*** / isolate NH59	***JN189598.1*** / isolate NH59 AB575125.1/ TNS:766491 DDU05622.1/ -
***Dryopteris dehuaensis* Ching & K. H. Shing**				DQ191838.1/ SG Lu/O9			
***Dryopteris erythrosora* (D. C. Eat.) O. Kuntze**		***KM208819.1*** / - PSMT3776/ - EF590692.1/ NMNH 06-8357	***JN189687.1*** / isolate NH13	***JN189255.1*** / isolate NH13 DQ191840.1/ SG Lu/B33	***AY268787.1*** / Geiger 94, COLO	***JN189361.1*** / isolate NH13	***AY268852.1*** / - ***AB232392.1***/ Tsutsumi CT1001 AY587111.1/ - KC896551.1/ isolate ERY 2006899
***Dryopteris expansa* (C. Presl) Fraser-Jenk. & Jermy**	JQ941606.1/ EBS 37 (WIS) JQ941612.1/ EBS 40 (WIS) JQ941633.1/ EBS 30 (WIS) JQ941610.1/ EBS 33 (WIS)	***AB575750.1*** / TNS:765180	***JN189616.1*** / isolate E064	***JN189180.1*** / isolate E064 KF020383.1/ Nelson 7921	***AY268775.1*** / Nelson 7921	***JN189291.1*** / isolate E064	***JQ935275.1*** / EBS 37 (WIS) EF463179.1/ Christenhusz 4263 (TUR) AB575127.1/ TNS:765180 AB908270.1/ -
***Dryopteris filix-mas* (L.) Schott**	JQ941618.1/ EBS 38 (WIS) JQ941611.1/ EBS 32 (WIS)	***JN189398.1*** / isolate E066	***JN189617.1*** / isolate E066	***JN189181.1*** / isolate E066	***FR731980.1*** / isolate A213	***JQ683060.1*** / EBS 38 (WIS)	***JN189507.1*** / isolate E066 JQ935265.1/ EBS 38 (WIS) KM114199.1/ Lehtonen 725 (TUR) JF832067.1/ -
***Dryopteris flaccisquama* A.Rojas**		***JN189411.1*** / isolate E236	***JN189629.1*** / isolate E236	***JN189193.1*** / isolate E236	***JN189088.1*** / isolate E236	***JN189305.1*** / isolate E236	***JN189519.1*** / -
***Dryopteris fragrans* (L.) Schott**	JQ941603.1/ EBS 47 (WIS)	JN189403.1/ isolate E081		JN189185.1/ isolate E081	AY268800.1/ Kelso 83-221	JQ683049.1/ EBS 53 (WIS) JN189297.1/ isolate E081	AB575129.1/ TNS:743728 JN189512.1 / isolate E081 JQ935274.1/ EBS 53 (WIS) AY268865.1/ -
***Dryopteris formosana* (Christ) C.Chr.**		***AB575751.1*** / TNS:763153	***JN189653.1*** / isolate E323	***JN189219.1*** / isolate E323	***AY268793.1*** / R. Moran, COLO	***JN189331.1*** / isolate E323	***JN189545.1*** / - AB575128.1/ TNS:763153 AY268857.1/ -
***Dryopteris fuscipes* C.Chr.**		AB575752.1/ TNS:762541		DQ191841.1/ SG Lu/M2	KC896583.1/ FUS 1058		AB575130.1/ TNS:762541 KC896549.1 \ isolate FUS 1058
***Dryopteris future* A.R.Sm.**		***JN189426.1*** / isolate E299	***JN189643.1*** / isolate E299	***JN189208.1*** / isolate E299	***JN189103.1*** / isolate E299	***JN189320.1*** / isolate E299	***JN189534.1*** / -
***Dryopteris goldiana* (Hook.) A.Gray**		***JN189396.1*** / isolate E063	***JN189615.1*** / isolate E063	***JN189179.1*** / -	***FR731984.1*** / isolate A202	***JN189290.1*** / isolate E063	***AF537228.1*** / -
***Dryopteris goeringiana* (G. Kze.) Koidz.**		***JN189474.1*** / isolate NH16	***JN189688.1*** / isolate NH16	***JN189256.1*** / -	***AY268790.1*** / - AF515241.1/ -	***JN189362.1*** / isolate NH16	***AY268855.1*** / R. Moran; COLO», cultivated in NY botanical garden KC896541.1/ isolate GOE 080368
***Dryopteris gymnosora* (Mak.) C. Chr.**		***JN189438.1*** / isolate E324	***JN189654.1*** / isolate E324	***JN189220.1*** / isolate E324 JX535824.1/ -	***JN189115.1*** / isolate E324 JX535877.1/ -	***JN189332.1*** / isolate E324	***AB575132.1*** / TNS:763325
***Dryopteris guanchica* Gibby & Jermy**		***JN189463.1*** / isolate E373	***JN189678.1*** / isolate E373	***JN189246.1*** / -	***FR731992.1*** / isolate A207	***JN189352.1*** / isolate E373	***JN189573.1*** / isolate E373
***Dryopteris hawaiiensis* (Hillebr.) Robinson**		***JN189470.1*** / isolate MA85	***JN189685.1*** / isolate MA85	***JN189253.1*** / isolate MA85	***JN189144.1*** / isolate MA85 AY268784.1/ Geiger 74, COLO	***JN189359.1*** / isolate MA85	***AY268840.1*** / -
***Dryopteris hondoensis* Koidz.**		***AB575761.1*** / TNS:764343	***JN189689.1*** / isolate NH17	***JN189257.1*** / isolate NH17	***AY268791.1*** / R. Moran, COLO	***JN189363.1*** / isolate NH17	***JN189583.1*** / isolate NH17 AB575139.1/ TNS:764343
***Dryopteris inaequalis* (Schlecht.) Kuntze**		***JN189440.1*** / isolate E326	***JN189655.1*** / isolate E326	***JN189222.1*** / isolate E326	***JN189117.1*** / isolate E326	***JN189333.1*** / isolate E326	***JN189548.1*** / isolate E326
***Dryopteris intermedia* (Willd.) A.Gray**	JQ941657.1/ EBS 63 (WIS) JQ941659.1/ EBS 44 (WIS) JQ941642.1/ EBS 69 (WIS) JQ941663.1/ EBS 13 (WIS) JQ941624.1/ ESB 15 (WIS) JQ941630.1/ EBS 18 (WIS)	***JQ936670.1*** / EBS 63 (WIS)	***JN189613.1*** / isolate E056	***JN189178.1*** / isolate E056	***FR731994.1*** / Alanko 96215 (H:1695737) FR731993.1/ isolate A141	***JN189288.1*** / isolate E056	***AB575143.1*** / TNS:766638
***Dryopteris juxtaposita* Christ**		***JN189441.1*** / isolate E327	***JN189656.1*** / isolate E327	***JN189223.1*** / isolate E327 DQ191848.1/ SG Lu/66	***JN189118.1*** / isolate E327 AY268810.1/ -	***JN189334.1*** / isolate E327	***JN189549.1*** / isolate E327 AY268875.1/ -
***Dryopteris karwinskyana* (Mett.) Kuntze**		***JN189413.1*** / isolate E241	***JN189631.1*** / isolate E241	***JN189195.1*** / isolate E241	***JN189090.1*** / isolate E241	***JN189307.1*** / isolate E241	***JN189521.1*** / isolate E241
***Dryopteris komarovii* Kossinsky**		***JN189442.1*** / isolate E328	***JN189657.1*** / isolate E328	***JN189224.1*** / isolate E328	***JN189119.1*** / isolate E328	***JN189335.1*** / isolate E328	***JN189550.1*** / -
***Dryopteris knoblochii* A. R. Sm.**		***JN189414.1*** / isolate E242	***JN189632.1*** / isolate E242	***JN189196.1*** / isolate E242	***JN189091.1*** / isolate E242	***JN189308.1*** / isolate E242	***JN189522.1*** / -
***Dryopteris kobayashii* Kitag.**							AB908374.1/ haplotype: Type 6
***Dryopteris labordei* (Christ) C.Chr.**		***JN189492.1*** / isolate NH84	***JN189709.1*** / isolate NH84	***JN189277.1*** / isolate NH84	***JN189169.1*** / isolate NH84	***JN189383.1*** / isolate NH84	***JN189602.1*** / isolate NH84 EF463128.1/ -
***Dryopteris lacera* (Thunb.) Ktze.**		***AB575770.1*** / TNS:774850	***JN189691.1*** / isolate NH23	***JN189259.1*** / isolate NH23 DQ191851.1/ SG Lu/QC36	***AY268794.1*** / R. Moran, COLO KC896572.1/ isolate LAC 1027	***JN189365.1*** / isolate NH23	***JN189585.1*** / isolate NH23 KC896538.1/ isolate LAC 1027
***Dryopteris ludoviciana* (Kze.) Small**	JQ941656.1/ EBSlud2 (WIS) JQ941628.1/ EBSlud3 (WIS) JQ941639.1/ EBSlud4 (WIS) JQ941654.1/ EBS 48.A (WIS)	***JQ936667.1*** / EBSlud2 (WIS)	***JQ947929.1*** / EBSlud4 (WIS)	***JN189186.1*** / isolate E082 JQ936837.1/ EBS 48 (WIS)	***JQ682979.1*** / EBSlud2 (WIS)	***JN189298.1*** / isolate E082	***JQ935277.1*** / EBSlud3 (WIS) JQ935273.1/ EBS 48 (WIS)
***Dryopteris marginalis* (L.) A.Gray**	JQ941607.1/ EBS 17 (WIS)	***JN189393.1*** / isolate E055	***JN189612.1*** / isolate E055	***JN189177.1*** / isolate E055	***FR731986.1*** / isolate A203	***JN189287.1*** / isolate E055	***EF463181.1*** / - KF186511.1/ OAC 96835
***Dryopteris maxonii* Underw. & C.Chr.**		***JN189421.1*** / isolate E284	***JN189639.1*** / isolate E284	***JN189203.1*** / isolate E284	***JN189098.1*** / isolate E284	***JN189315.1*** / isolate E284	***JN189529.1*** / isolate E284
***Dryopteris medioxima* Koidz.**		AB575774.1/ TNS:776976					AB575152.1/ 776976
***Dryopteris munchii* A.R.Sm.**	JQ941632.1/ EBS 54 (WIS)	***JN189427.1*** / isolate E311	***JN189644.1*** / isolate E311	***JN189209.1*** / - JQ936827.1/ Hoshizaki (UC)	***AY268822.1*** / -	***JN189321.1*** / WIS EBS54	***JN189535.1*** / isolate E311
***Dryopteris odontoloma* (Moore) C. Chr.**		***JN189483.1*** / isolate NH46	***JN189696.1*** / isolate NH46	***JN189265.1*** / - DQ191859.1/ -	***AY268807.1*** / -	***JN189371.1*** / isolate NH46	***JN189590.1*** / isolate NH46 AY268872.1/ -
***Dryopteris oligodonta* (Desv.) Pichi-Serm.**	JQ941643.1/ voucher Hennequin 2010-C11 (P)	***JN189464.1*** / isolate E374	***JN189679.1*** / isolate E374	***JN189247.1*** / isolate E374	***FR731987.1*** / isolate A156	***JN189353.1*** / isolate E374	***JN189574.1*** / isolate E374
***Dryopteris pacifica* (Nakai) Tag.**		***AB575778.1*** / TNS:763312	***JN189700.1*** / isolate NH53	***JN189269.1*** / isolate NH53 DQ191860.1/ - JX535838.1/ -	***JX535891.1*** / - KC896588.1/ isolate PAC 487 AY268814.1/ -	***JN189375.1*** / isolate NH53	***JN189594.1*** / - AB575157.1/ TNS:763312
***Dryopteris panda* (C. B. Cl.) C. Chr.**		***JN189443.1*** / isolate E329	***JN189658.1*** / isolate E329	***JN189225.1*** / - DQ191861.1/ -	***JN189120.1*** / isolate E329	***JN189336.1*** / isolate E329	***JN189551.1*** / -
***Dryopteris patula* (Sw.) Underw.**		***JN189391.1*** / isolate E047	***JN189610.1*** / isolate E047	***KJ464668.1*** / isolate MS1104	***AY268823.1*** / -	***JN189285.1*** / isolate E047	***KJ464427.1*** / - AY268888.1 -
***Dryopteris pentheri* (Krasser) C. Chr.**		***JN189466.1*** / isolate E377	***JN189681.1*** / isolate E377	***JN189249.1*** / isolate E377	***JN189140.1*** / isolate E377	***JN189355.1*** / isolate E377	***KF992481.1*** / -
***Dryopteris polita* Rosenst.**	KJ196593.1/ S.Y. Dong 718	***AB575779.1*** / TNS:763901	***JN189713.1*** / isolate NH92	***JN189281.1*** / isolate NH92	***KJ196700.1*** / S.Y. Dong 718	***JN189387.1*** / isolate NH92	***AB575158.1*** / TNS:763901
***Dryopteris polylepis* (Franch. & Sav.) C. Chr.**		***AB575780.1*** / TNS:764340	***JN189695.1*** / isolate NH33	***JN189263.1*** / isolate NH33	***AY268798.1*** / R. Moran, COLO	***JN189369.1*** / isolate NH33	***AB575159.1*** / TNS:764340
***Dryopteris pseudofilix-mas* (Fée) Rothm.**		***JN189415.1*** / isolate E259	***JN189633.1*** / isolate E259	***JN189197.1*** / isolate E259	***AY278404.1*** / -	***JN189309.1*** / isolate E259	***JN189523.1*** / -
***Dryopteris pulcherrima* Ching**		***JN189406.1*** / isolate E108	***JN189624.1*** / isolate E108	***JN189188.1*** / isolate E108	***JN189083.1*** / isolate E108 AY268811.1/ -	***JN189300.1*** / isolate E108	***JN189515.1*** / isolate E108
***Dryopteris pycnopteroides* (Christ) C. Chr.**		***AB575781.1*** / TNS:764373	***JN189690.1*** / isolate NH22	***JN189258.1*** / isolate NH22 DQ191868.1/ SG Lu/C79	***AY268799.1*** / R. Moran, COLO	***JN189364.1*** / isolate NH22	***AB575160.1*** / TNS:764373 AY587114.1/ -
***Dryopteris remota* Hayata**	JQ941635.1/ Schuettpelz 528 (DUKE) JQ941616.1/ Moran.A (COLO)	***JQ936655.1*** / Moran (COLO)	***JN189640.1*** / isolate E285	***JN189204.1*** / Isolate E285 JQ936826.1/ Moran (COLO)	***JQ682983.1*** / Moran (COLO) AY268792.1/ R. Moran, COLO	***JN189316.1*** / isolate E285 JQ683062.1/ Moran (COLO)	***AY268858.1*** / - JQ935253.1/ Moran (COLO)
***Dryopteris reflexosquamata* Hayata**		***JN189494.1*** / isolate NH86	***JN189711.1*** / isolate NH86	***JN189279.1*** / isolate NH86	***JN189171.1*** / isolate NH86	***JN189385.1*** / isolate NH86	***JN189604.1*** / isolate NH86
***Dryopteris rosea* (E.Fourn.) Mickel & Beitel**		***JN189416.1*** / isolate E260	***JN189634.1*** / isolate E260	***JN189198.1*** / isolate E260	***JN189093.1*** / isolate E260	***JN189310.1*** / isolate E260	***JN189524.1*** / -
***Dryopteris rossii* C.Chr.**		***JN189423.1*** / isolate E286	***JN189641.1*** / isolate E286	***JN189205.1*** / isolate E286	***JN189100.1*** / isolate E286	***JN189317.1*** / isolate E286	***JN189531.1*** / isolate E286
***Dryopteris sacrosancta* Koidz.**		***AB575784.1*** / TNS:764377	***JN189698.1*** / isolate NH51	***JX535843.1*** / cult. Tsukuba Botanical Garden; VS764377 (TNS) JN189267.1/ AFSSE	***JX535896.1*** / cult. Tsukuba Botanical Garden; VS764377 (TNS) AY268812.1/ -	***JN189373.1*** / isolate NH51	***AY268877.1*** / - AB575163.1/ TNS:764377
***Dryopteris salvinii* (Bak.) Kuntze**		***JN189418.1*** / isolate E264	***JN189636.1*** / isolate E264	***JN189200.1*** / isolate E264	***JN189095.1*** / isolate E264	***JN189312.1*** / isolate E264	***JN189526.1*** / isolate E264
***Dryopteris scottii* (Bedd.) Ching**	JQ941623.1/ voucher RBC 202 (UC)	***JN189444.1*** / isolate E330	***JN189659.1*** / isolate E330	***JN189226.1*** / iIsolate E330 DQ191872.1/ SG Lu/B31	***JX535898.1*** / Hai He 4 (CTC) DQ514498.1/ MMO03-313	***JN189337.1*** / isolate E330	***JX535863.1*** / - DQ508775.1/ -
***Dryopteris simplicior* Mickel & Beitel**		***JN189419.1*** / isolate E265	***JN189637.1*** / isolate E265	***JN189201.1*** / isolate E265	***JN189096.1*** / isolate E265	***JN189313.1*** / isolate E265	***JN189527.1*** / isolate E265
***Dryopteris sordidipes* Tagawa**		***JN189495.1*** / isolate NH88 AB575793.1/ TNS:763050	***JN189712.1*** / isolate NH88	***JN189280.1*** / isolate NH88 JX535848.1/ S. Tagane & K. Fuse TF009; VS763050 (TNS)	***JX535902.1*** / S. Tagane & K. Fuse TF009; VS763050 (TNS)	***JN189386.1*** / isolate NH88	***AB575172.1*** / TNS:763050
***Dryopteris stenolepis* (Baker) C.Chr.**		***JN189445.1*** / isolate E331	***JN189660.1*** / isolate E331	***JN189227.1*** / isolate E331 DQ191877.1/ SG Lu/B28	***JN189122.1*** / isolate E331 AY268824.1/ -	***JN189338.1*** / isolate E331	***AY268889.1*** / -
***Dryopteris stewartii* Fraser-Jenk.**		***JN189478.1*** / isolate NH26	***JN189692.1*** / isolate NH26	***JN189260.1*** / isolate NH26	***AY278405.1*** / -	***JN189366.1*** / isolate NH26	***JN189586.1*** / isolate NH26
***Dryopteris subbipinnata* W.H.Wagner & R.W.Hobdy**		***JN189469.1*** / isolate MA116	***JN189684.1*** / isolate MA116	***JN189252.1*** / isolate MA116	***AY268765.1*** / Oppenheimer H50074, COLO	***JN189358.1*** / isolate MA116	***JN189579.1*** / isolate MA116
***Dryopteris sublacera* Christ**		***JN189446.1*** / isolate E332	***JN189661.1*** / isolate E332	***JN189228.1*** / isolate E332 DQ191878.1/ SG Lu/59	***JN189123.1*** / isolate E332 AY268788.1/ Geiger 95, COLO DQ514501.1/ LJM080	***JN189339.1*** / isolate E332	***JN189554.1*** / isolate E332
***Dryopteris tokyoensis* (Matsum.) C.Chr.**	JQ941651.1/ voucher Moran.B (COLO)	***AB575795.1*** / TNS:766452	***JN189683.1*** / isolate JGtok	***JN189251.1*** / isolate JGtok	***AY268795.1*** / R. Moran, COLO	***JN189357.1*** / isolate JGtok	***AB575174.1*** / TNS:766452
***Dryopteris uniformis* Makino**		***AB575797.1*** / TNS:774834	***JN189662.1*** / isolate E333	***DQ191883.1*** / -	***AY268806.1*** / -	***JN189340.1*** / isolate E333	***JN189555.1*** / isolate E333
***Dryopteris varia* (L.) Kuntze**		***AB575798.1*** / TNS:763911	***JN189663.1*** / isolate E334	***JN189230.1*** / - JX535852.1/ -	***AY736355.1*** / -	***JN189341.1*** / isolate E334	***AB575178.1*** / TNS:763911
***Dryopteris wallichiana* (Spreng.) Hyl.**		***JN189388.1*** / -	***JN189607.1*** / isolate E001	***DQ191884.1*** / -	***AY268761.1*** / -	***JN189282.1*** / isolate E001	***AY268826.1*** / - KF992482.1/ S. Hennequin 288 (P REU)
***Dryopteris xanthomelas* (Christ) C.Chr.**		***JN189449.1*** / -	***JN189665.1*** / isolate E337	***JN189232.1*** / -	***JN189127.1*** / -	***JN189343.1*** / isolate E337	***JN189558.1*** / isolate E337
**Dryopteris blanfordii (C.Hope) C.Chr. subsp. nigrosquamosa (Ching) Fraser-Jenk.**	KT876448.1/ -	***KT876440.1*** / -	***KT876441.1*** / -	***KT876442.1*** / -	***KT876443.1*** / -	***KT876444.1*** / -	***KT876447.1*** / -
***Polystichum andersonii* Hopkins**	JQ941662.1/ EBS 39 (WIS)	***JN189401.1*** / isolate E073	***JN189620.1*** / isolate E073	***JN189183.1*** / isolate E073	***JN189078.1*** / isolate E073	***JN189295.1*** / isolate E073	***JN189510.1*** / isolate E073
